# Zinc supplementation modifies brain tissue transcriptome of *Apis mellifera* honeybees

**DOI:** 10.1186/s12864-022-08464-1

**Published:** 2022-04-08

**Authors:** Marcelo Polizel Camilli, Samir Moura Kadri, Marcus Vinícius Niz Alvarez, Paulo Eduardo Martins Ribolla, Ricardo Oliveira Orsi

**Affiliations:** 1 Center of Education, Science and Technology in Rational Beekeeping (NECTAR), College of Veterinary Medicine and Animal Sciences, UNESP São Paulo State University, São Paulo, Botucatu, Brazil; 2 Institute of Biotechnology, UNESP - São Paulo State University, São Paulo, Botucatu, Brazil

**Keywords:** Apis mellifera, Zinc, Gene expression, Nutrigenomics

## Abstract

**Background:**

Bees are the most important group of pollinators worldwide and their populations are declining. In natural conditions, *Apis mellifera* depends exclusively on food from the field to meet its physiological demands. In the period of scarcity, available resources are insufficient and artificial supplementation becomes essential for maintaining the levels of vitamins, proteins, carbohydrates, and minerals of colonies. Among these minerals, zinc is essential in all living systems, particularly for the regulation of cell division and protein synthesis, and is a component of more than 200 metalloenzymes.

**Results:**

The total RNA extracted from the brain tissue of nurse bees exposed to different sources and concentrations of zinc was sequenced. A total of 1,172 genes in the treatment that received an inorganic source of zinc and 502 genes that received an organic source of zinc were found to be differentially expressed among the control group. Gene ontology enrichment showed that zinc can modulate important biological processes such as nutrient metabolism and the molting process.

**Conclusions:**

Our results indicate that zinc supplementation modulates the expression of many differentially expressed genes and plays an important role in the development of *Apis* mellifera bees. All the information obtained in this study can contribute to future research in the field of bee nutrigenomics.

**Supplementary Information:**

The online version contains supplementary material available at 10.1186/s12864-022-08464-1.

## Background

Bees are the most important group of pollinators in the world that visit90% of the major107 global crop types and assist the maintenance of ecosystems [1]. *Apis mellifera* honeybees are the most commonly managed species, which provide high-value pollination services for agricultural crops [1]. Recent declines in honeybees population have been attributed to multiple interacting stressors, of which, the most important are nutrition deficits, pesticides, parasites, and pathogens [2].

Under natural conditions, *Apis mellifera* bees depend exclusively on two food sources to supply colonies: nectar as a source of energy and pollen as a source of protein, vitamins, and minerals [3, 4]. Minerals are needed to maintain life because they play significant roles as structural components and enzymatic cofactors [5]. Among the essential minerals, some are distinct for participating in important metabolic pathways, such as zinc [6].

Zinc is the second most essential element in all living systems and is essential for the regulation of cell division, protein synthesis, and DNA [6–8]. Zinc ions can penetrate the peritrophic membrane and midgut epithelial cells, and transporters for delivering zinc ions can be influenced by dietary Zn levels [7]. This microelement is transferred to the hemolymph, where vitellogenin acts as the main Zn transporter [8, 9]. Zinc is a component of more than 200 metalloenzymes and other metabolic compounds that modulate biochemical processes which involve the maintenance of cell membrane integrity, cellular respiration and reproduction, and other essential functions such as binding nucleic acids as a zinc finger complex [10].

Zinc cannot be stored in the body [11] and requires regular dietary intake to meet physiological needs. Thus, zinc is routinely supplemented in human and livestock foods and feeds for normal physiological functions as well as to meet daily requirements [12]. Zinc is commonly supplemented in two forms: organic form (methionine) and inorganic form (sulfate); minerals linked to organic molecules called chelates have advantages over the inorganic form, with greater absorption and less competition for binding sites with other minerals [13]. However, few studies have clarified the nutritional requirements of *Apis mellifera* bees.

The advent of next-generation sequencing (NGS) technology has revolutionized the way biological research is conducted [14]; in this study, RNA-seq was used to investigate the effects of zinc supplementation at different dosages and sources on the transcriptome profiles of *A. mellifera* bees.

## Results and Discussion

### Sequencing quality and Differential Gene Expression (DGE) library sequencing

DGE tag libraries were constructed and sequenced using total RNA extracted from the brain tissue of *A. mellifera* nursing bees. For each library, brain tissue was dissected from five worker bees and pooled as a sample to construct the library.

The sequencing resulted in a total of 352,957,663 raw sequences. The sequences were analyzed using FastQC program. No adapter content was identified. The global mean sequence quality was 36 (Phred Score), and the mean sequence length was 76 bp. The per tile quality was excellent and no variations were identified. The %GC content observed was 40%. No sequence was flagged as poor quality. The mean per base sequence quality from base 1 to base 74 was 34.26, with minimum of 30.95 at the 76th base and maximum of 35.2 at the 6th base. The sequence alignment resulted in a mean mapping rate of 96.15% (Table 1).

**Table 1:** Sequencing data quality

Sample	Raw total sequences	Readsmapped	Readsunmapped	Averagequality	Averagelength	Error rate
Znctl-1	14,403,139	14,265,441	137,698	34,3	75	0,00,849,035
Znctl-2	21,458,852	12,170,485	9,288,367	32,2	75	0,00,701,732
Znctl-3	13,585,841	13,434,076	151,765	34,4	75	0,00,862,269
Znctl-4	24,119,701	23,874,376	245,325	34,4	75	0,01,052,767
ZnI25-1	11,711,531	11,593,242	118,289	34,4	75	0,00,879,498
ZnI25-2	1,016,724	1,002,612	14,112	34,3	75	0,01,013,923
ZnI25-3	14,096,419	13,647,316	449,103	34,2	75	0,01,010,243
ZnI25-4	15,432,743	15,107,742	325,001	34,3	75	0,01,026,254
ZnI50-1	10,046,130	9,947,956	98,174	34,3	75	0,0,092,947
ZnI50-2	11,712,178	11,645,390	66,788	34,4	75	0,00,943,242
ZnI50-3	10,647,007	10,483,311	163,696	34,4	75	0,01,001,656
ZnI50-4	6,532,054	6,478,483	53,571	34,4	75	0,00,980,485
ZnI75-1	6,231,565	6,180,502	51,063	34,4	75	0,00,996,597
ZnI75-2	13,291,595	11,770,621	1,520,974	33,7	75	0,00,902,715
ZnI75-4	8,357,610	7,685,140	672,470	34,2	75	0,00,992,565
ZnO25-1	19,652,161	19,524,530	127,631	34,4	75	0,0,097,472
ZnO25-2	14,979,054	14,688,229	290,825	34,4	75	0,00,878,063
ZnO25-3	10,887,356	10,806,772	80,584	34,4	75	0,00,999,203
ZnO25-4	9,482,014	9,215,969	266,045	34,3	75	0,00,900,701
ZnO50-1	30,808,010	30,430,020	377,990	34,4	75	0,00,947,306
ZnO50-2	8,750,930	8,675,284	75,646	34,4	75	0,01,025,609
ZnO50-3	9,829,283	9,741,018	88,265	34,4	75	0,01,044,157
ZnO50-4	10,374,188	9,401,726	972,462	34,2	75	0,0,115,602
ZnO75-1	17,128,454	16,818,111	310,343	34,4	75	0,00,972,479
ZnO75-2	9,231,895	9,021,197	210,698	34,3	75	0,00,934,483
ZnO75-3	17,231,058	16,989,001	242,057	34,4	75	0,01,015,037
ZnO75-4	11,960,171	11,773,429	186,742	34,3	75	0,00,981,345

The sequencing results showed that the four biological replicates of each sample had high reproducibility with high expression correlation value (Figure S1), suggesting the high reliability of the sequencing results (Table S1).

### Analysis of gene expression of bees supplemented with different sources of zinc

**Fig. 1:**
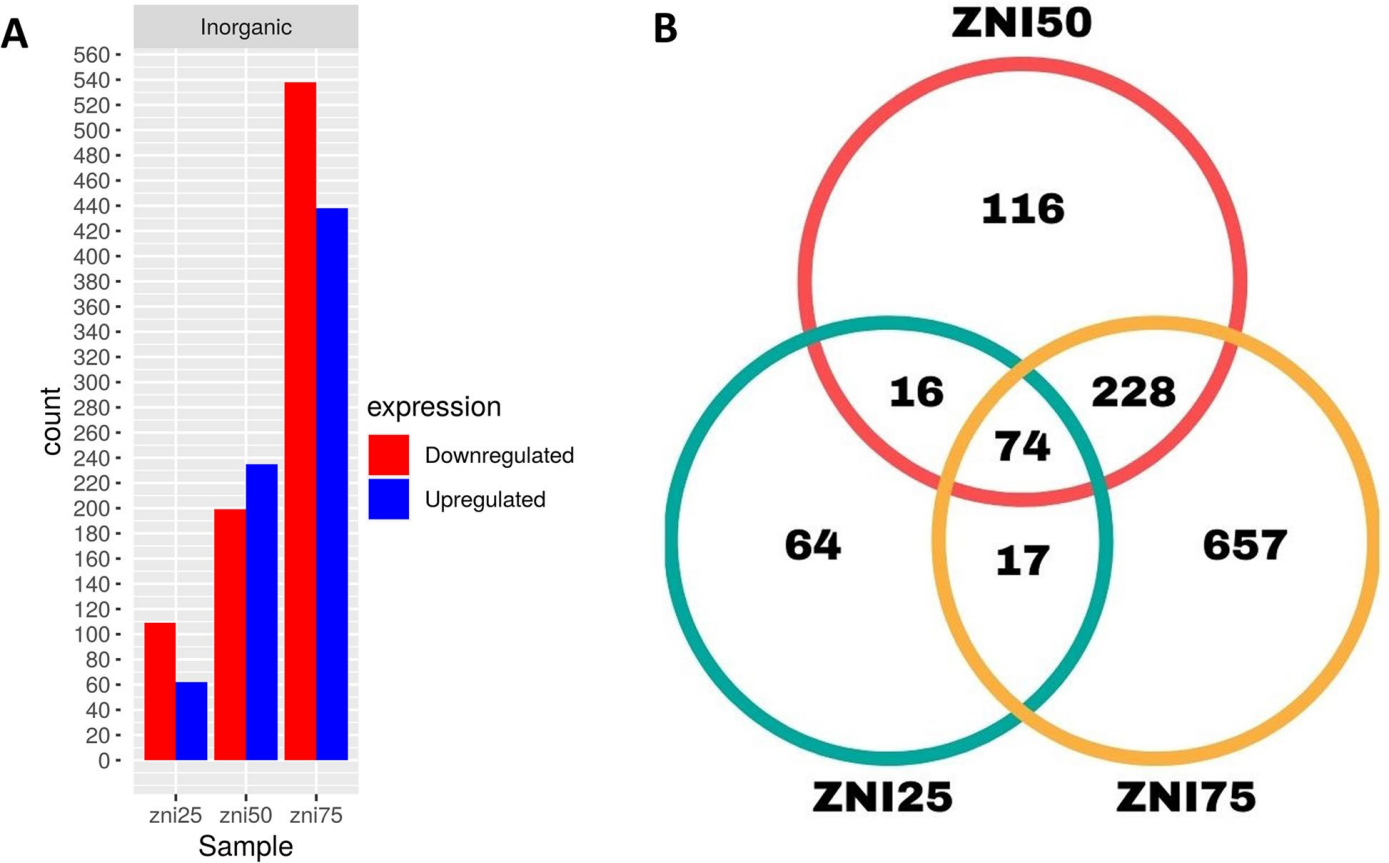
Differentially expressed genes (DEGs) between experimental groups (ZnI25, ZnI50, and ZnI75) compared to control group Zn0. (**A**) Histogram of DEGs between experimental groups supplemented with inorganic source of zinc at each concentration of zinc (25 ppm, 50 ppm, and 75 ppm). (**B**) Venn diagram of DEGs between experimental groups supplemented with inorganic source of zinc at each concentration of zinc

After supplementing the bees with different concentrations of inorganic zinc, the treatments showed 1,172 differentially expressed genes when compared to the control, with theZnI75 group having the highest number of upregulated and downregulated genes, followed by ZnI50 and ZnI25, which had the lowest number of expressed genes. Of these differentially expressed genes, 64, 116, and 657 were exclusively expressed in the ZnI25, ZnI50, and ZnI75 groups, respectively, and 74 genes were expressed for the three treatments, with the treatment with 75 ppm having a higher number of modulated genes (Fig. 1). As we increased the dose of inorganic zinc in the diet, gene expression also increased. However, as shown in Fig. 1, the medium doses containing inorganic zinc (50 ppm) showed a higher number of upregulated genes compared to the downregulated ones, unlike the other dosages (25 and 75 ppm), which showed a lower number of upregulated genes.

Regarding the organic zinc supplementation, the treatments showed 502 genes that were differentially expressed when compared to the control, with the ZnO25 group having the highest number of upregulated and downregulated genes, followed by ZnO75 and ZnO50, which had the lowest number of expressed genes.

**Fig. 2:**
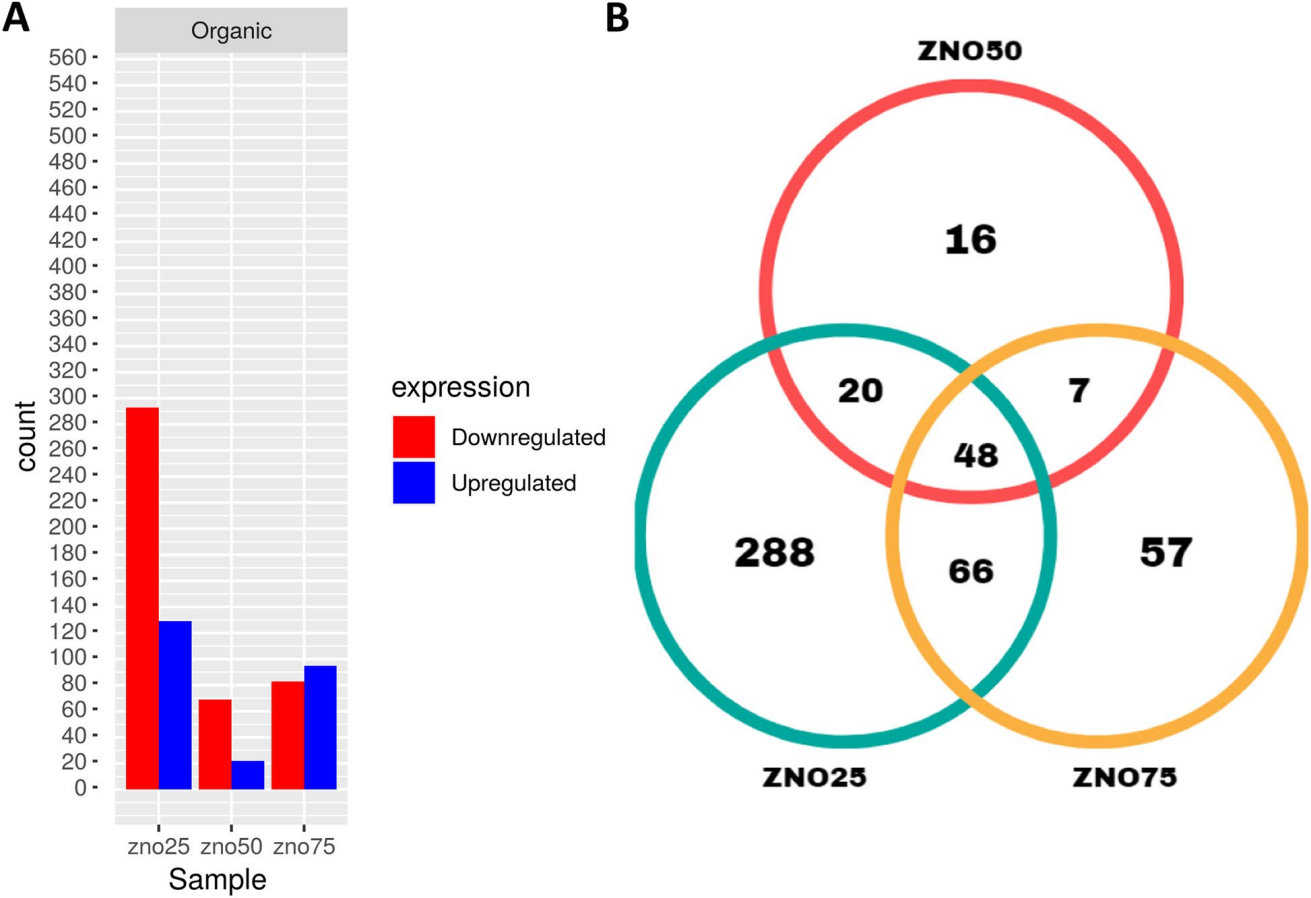
Differentially expressed genes (DEGs)between experimental groups (ZnO25, ZnO50, and ZnO75) compared to control group Zn0. (**A**) Histogram of DEGs between experimental groups supplemented with organic source of zinc at each concentration of zinc (25 ppm, 50 ppm, and 75 ppm). (**B**) Venn diagram of DEGs between experimental groups supplemented with inorganic source of zinc at each concentration of zinc

ZnO75 group having the highest number of upregulated genes and the ZnO25 and ZnO50 groups with the highest number of downregulated genes. Of these genes, 288, 16, and 57 genes were expressed exclusively in the ZnO25, ZnO50, and ZnO75 groups, respectively, and 48 genes were expressed in the three treatments, with the treatment with 25 ppm having a higher number of modulated genes (Fig. 2). Contrary to what was observed with inorganic zinc, the treatment with a higher dose of organic zinc in the supplementation of *A. mellifera* bees had a smaller number of modulated genes.

### Differentially expressed genes (DEGs) between treatments supplemented with organic and inorganic sources of zinc

**Fig. 3:**
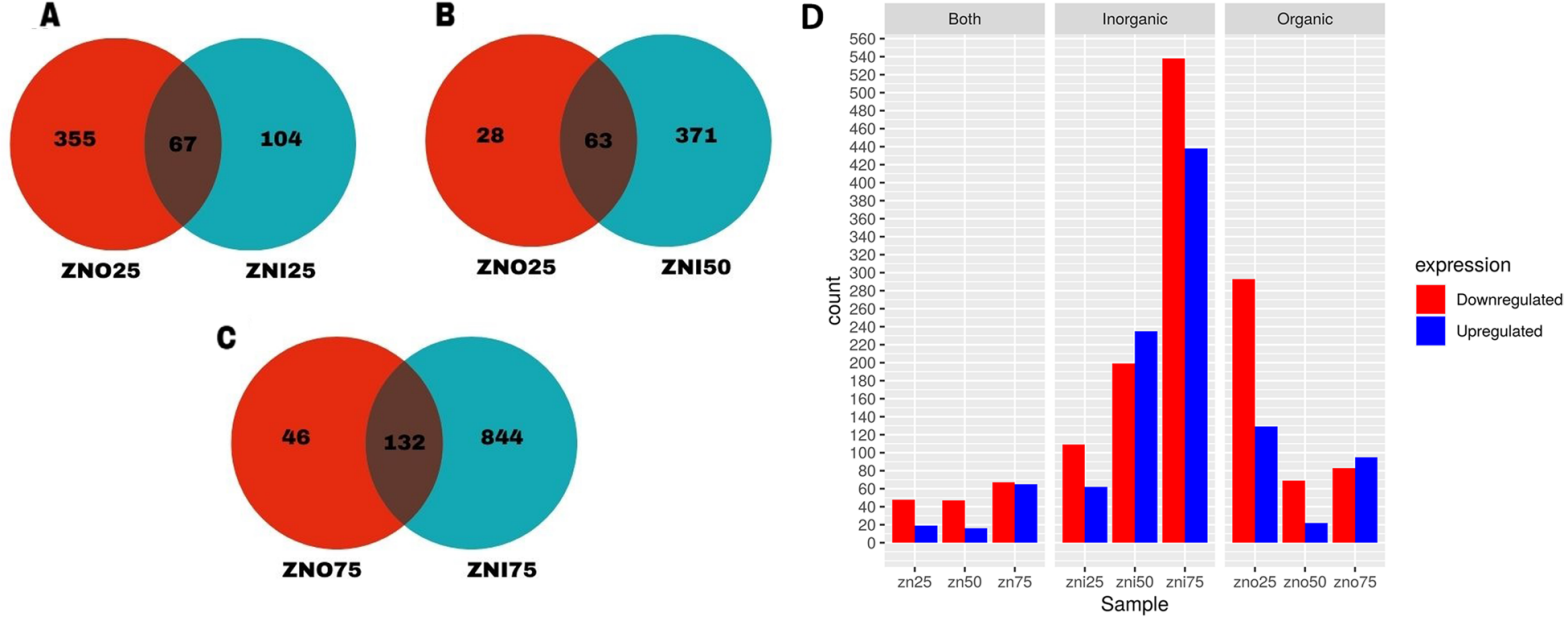
Differentially expressed genes (DEGs) between experimental groups supplemented with inorganic and organic source of zinc at each concentration of zinc. **A**) Venn diagram of DEGs between experimental groups ZnO25 and ZnI25 compared to control group Zn0.**B**) Venn diagram of DEGs between experimental groups ZnO50 and ZnI50 compared to control group Zn0. **C**) Venn diagram of DEGs between experimental groups ZnO75 and ZnI75 compared to control group Zn0. **D**) Histogram of DEGs between experimental groups supplemented with both groups, inorganic and organic source of zinc at each concentration of zinc

Based on a comparison of treatments supplemented with the same concentrations of zinc, but with different sources (organic vs. inorganic), we found that organic zinc showed higher gene expression only in the lowest concentration of zinc supplementation (25 ppm). In the other experimental groups (50 and 75 ppm), inorganic zinc showed higher gene expression when compared to treatments supplemented with zinc from organic sources (Fig. 3). In addition, the results suggest that an organic source of zinc at a lower concentration (25 ppm) can increase the gene expression of *A. mellifera*. Contrasting this, for inorganic zinc, the highest concentration (75 ppm) can increase the gene expression of honeybees.

According to the histogram, the gene expression of both groups has a predominance of down-regulated genes for all levels of zinc supplementation. When we observe separately, the group that received 75 ppm of inorganic zinc (ZnI75) had the greatest number genes expressed, being mostly downregulated genes, in the ZnI50 group we observed a greater number of upregulated genes compared to the downregulated genes, the ZnI25 group presented the lesser number of regulated genes with the greater number of downregulated genes. Regarding the groups that received organic zinc, the group that received25 ppm of inorganic zinc (ZnO25) had the greatest number genes expressed, being mostly downregulated genes, in the ZnO75 group we observed a greater number of upregulated genes compared to the downregulated genes, the ZnO50 group presented the lesser number of regulated genes with the greater number of downregulated genes.

### Gene Ontology enrichment analysis

In gene ontology (GO) analysis, when we compared the intersection between genes differentially expressed for the two sources of zinc (organic and inorganic), we observed that both sources stimulated genes related to important biological processes, such as chitin metabolism, amino acids, and amino glycans, as well as structural components of *A. mellifera* bee chitin (Figure S2).

Zinc is an essential mineral for all living beings [8], and in insects, it can be related to body growth. For insects to exchange their chitin exoskeleton and complete their development, the action of chitinase enzymes is necessary, whose function is to digest the polysaccharides that form chitin [15–17]. During the metamorphosis process, the chitinase BmCHT5 is expressed in *Bombyx mori* [18] and when a zinc finger element is present (BR–C Z4), the expression of this enzyme is improved. Therefore, the amount of zinc absorbed in bee nutrition can influence the action of these enzymes and, consequently, the process of molting these insects.

According to Keilling [19], zinc is a structural and catalytic component of hundreds of classes of enzymes and is highly related to the metabolism of nucleic acids and carbohydrates. In addition to affecting the functioning of digestive enzymes in rapidly growing tissues, zinc deficiency dramatically decreases the synthesis of RNA, DNA, and proteins, thereby decreasing the rate of mitosis and compromising tissue growth and recovery [11].

*A. mellifera* bees depend exclusively on two sources of nutrients, pollen and nectar. Pollen is composed of starch, vitamins, and minerals and depends on the action of amylase enzymes to be digested. Among these nutrients, zinc is essential for the regulation of amylase enzymes, and its function has been described in several animal species, such as pigs, rats, and chickens [20].

**Fig. 4:**
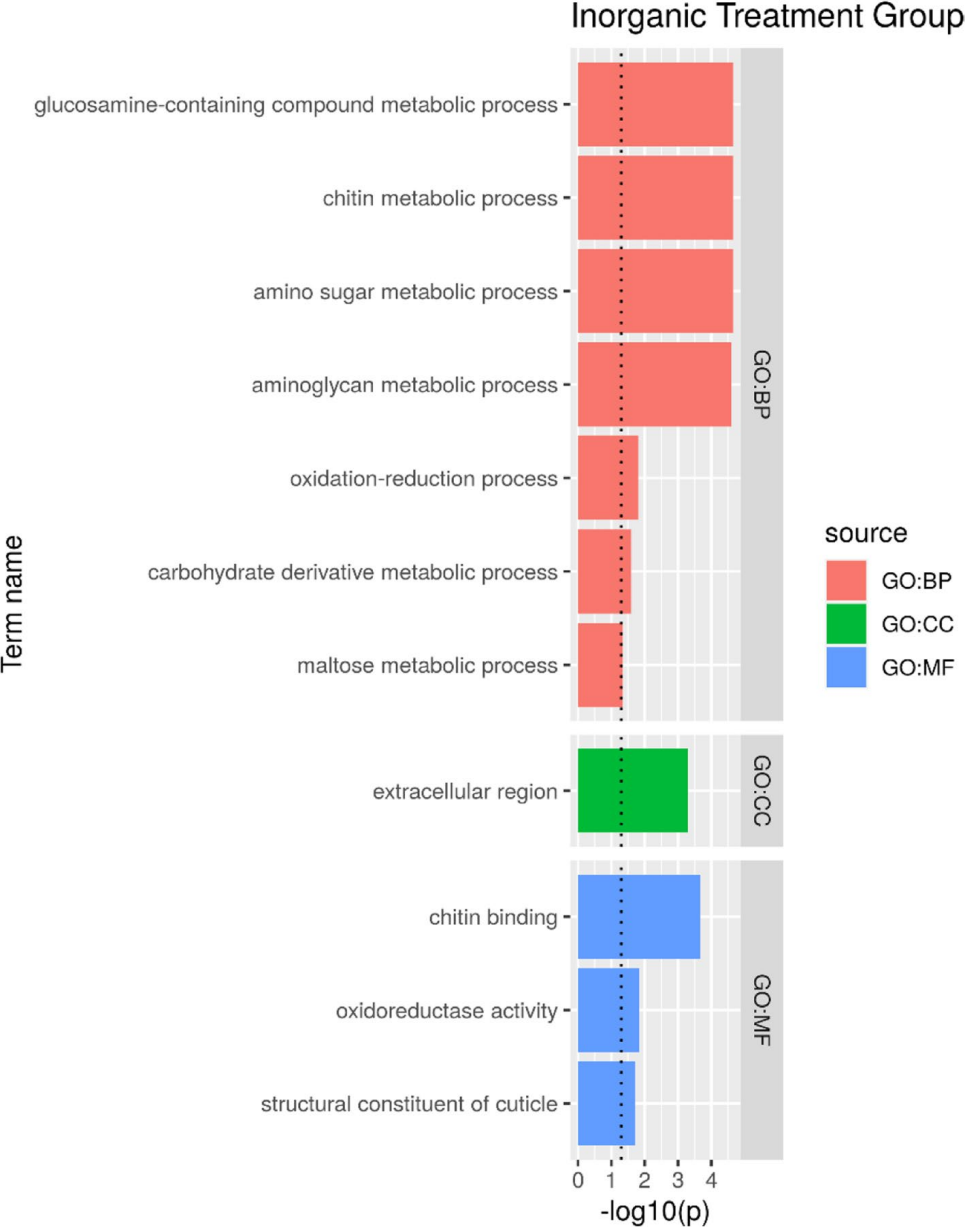
Gene ontology analysis of the experimental groups supplemented with inorganic source of zinc (ZnI25, ZnI50, and ZnI75). Red color indicates biological processes, blue color indicates molecular functions, and green color indicates cellular components

Inorganic zinc treatments showed1,172 differentially expressed genes, enabling the GO analysis. Our results suggest that the sources of inorganic zinc enriched the pathways related to important biological processes, such as drug and nutrient metabolism, cuticle development and metabolism, and processes related to arthropod ecdysis (Fig. 4).

Among the altered gene pathways, detoxification seems to be related to zinc supplementation. *A. mellifera* bees depend on a set of enzymes to metabolize drugs and pesticides, including cytochrome P450 monooxygenases [21]. Cytochrome P450 plays a role in the detoxification of phytochemicals present in the food consumed by bees [22–24]. Our results suggest that zinc participates in the detoxification process and may modulate the action of monooxygenase enzymes in bees.

We observed the modulation of genes related to the body development processes of bees. Hiki [25] analyzed the crustacean transcriptome and reported that exposure to inorganic zinc (zinc sulfate) dosages influenced genes related to the exoskeleton, thereby inhibiting the molting process in these arthropods.

**Fig. 5:**
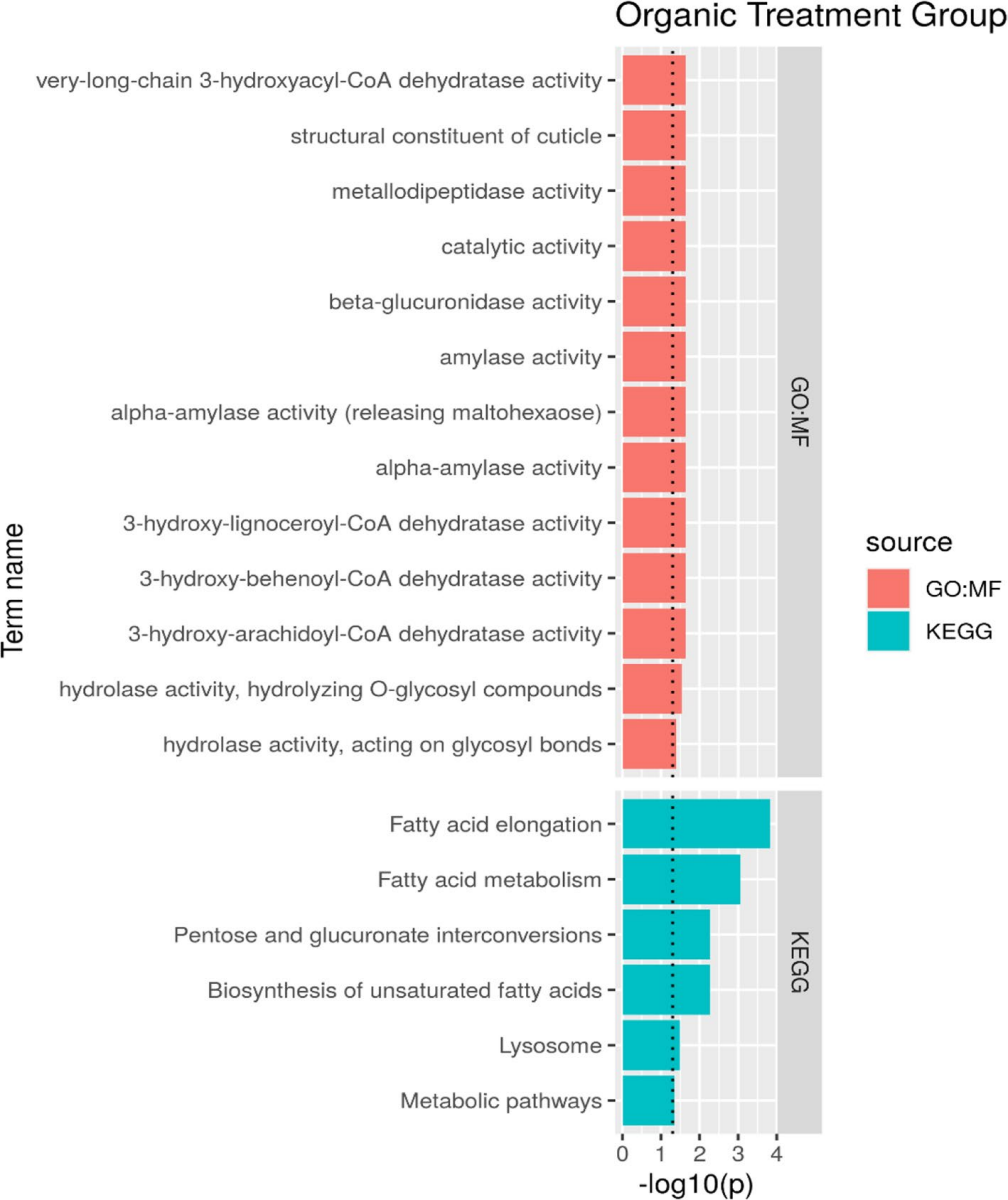
Gene ontology analysis of the experimental groups supplemented with organic source of zinc (ZnO25, ZnO50, and ZnO75). Red color indicates biological processes, i.e., molecular functions and the blue color indicates KEGG

For organic zinc treatments, we identified 502 differentially expressed genes, enabling the GO analysis. Our results suggest that the sources of organic zinc enriched the pathways related to important biological processes, such as nutrient metabolism and cuticle development (Fig. 5).

According to the GO analysis, organic zinc supplementation influenced specific pathways related to lipid metabolism. Organic zinc sources altered gene pathways related to the enzyme 3-hydroxyacyl-CoA dehydratase (DEH) activity, lysosome activity, and chitin binding. DEH is a key member of lipid metabolism [26] involved in very long chain fatty acid synthesis, which interacts with several elongases and plays an essential role during development, differentiation, and maintenance of a number of tissue types [27].

In insects such as *Musca* sp., a few lysosomes may occur and contain minerals such as Ca, Fe, Cu, P, and Zn with functions related to mineral detoxification. Insects deprived of organs with both spherocrystals and lysosomes are less able to resist mineral contamination [28]. According to the GO results, organic zinc supplementation may increase the activity of lysosomes, possibly allowing it to store the amount of zinc absorbed.

As observed in treatments that received zinc from an inorganic source, organic zinc also modulated the expression of genes related to chitin binding, suggesting a relationship between the process of ecdysis and the mineral zinc absorbed by *A. mellifera* honeybees.

**Fig. 6:**
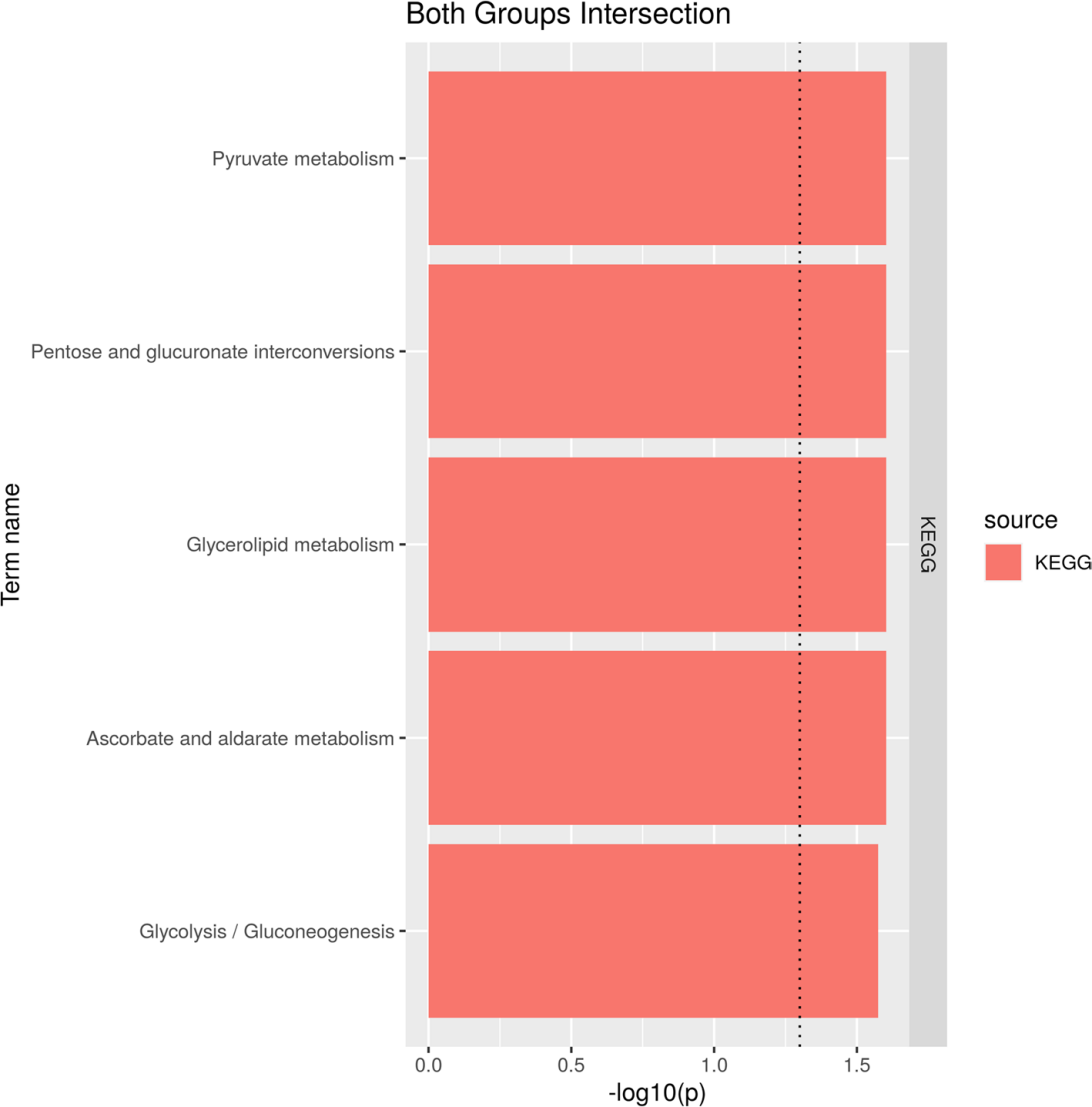
Gene ontology analysis of ZnO and ZnI groups interaction

Among the statistically enriched pathways in both treatments (Fig. 6), we obtained relationships with energy metabolism (Pyruvate, Glycerlipidio and Glyciolysis). Glycolipids are widely distributed in every tissue of the body, particularly in nervous tissue such as brain which depends on high energy demand to perform its functions [29]. Glycolysis and the oxidation of pyruvate and Ascorbate, is related to antioxidant metabolism pathways were Zinc is directly involved in antioxidant enzymes [30], which catalyze the decomposition of hydrogen peroxide (H_2_O_2_) in water, using ascorbate as an electron donor. H_2_O_2_ is a reactive oxygen species (ROS) produced by aerobic metabolism and in stressful situations [31]. Brain tissues are more vulnerable to oxidative stress than other insect tissues, as they consume much oxygen and contain large amounts of polyunsaturated fatty acids available for lipid peroxidation [32].

### Heat map of DGE from experimental groups

**Fig. 7:**
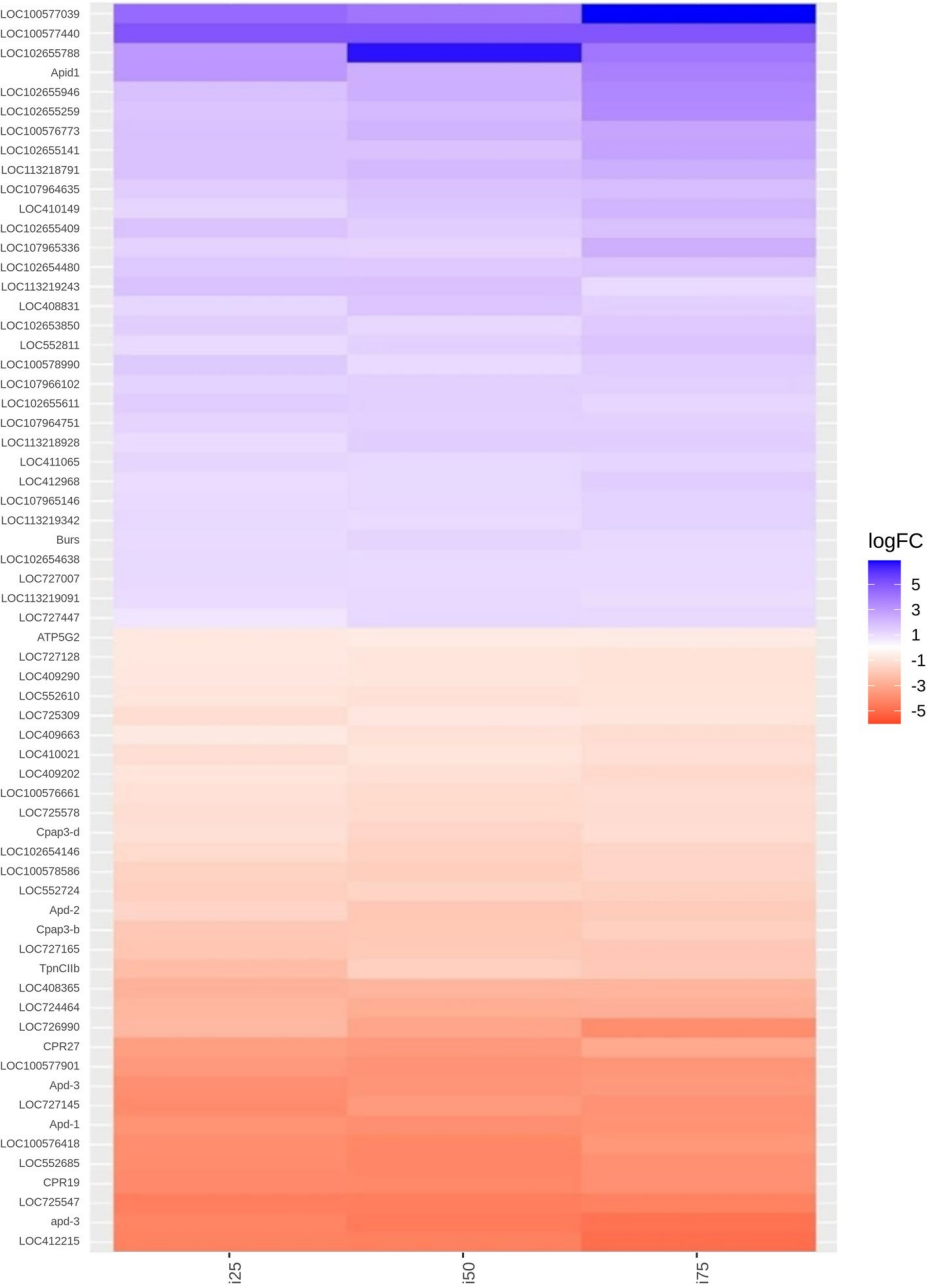
Clustering analysis of the differentially expressed genes between experimental groups supplemented with inorganic source of zinc and control group

The heatmap graph (Fig. 7) shows that the colonies that received dosages of inorganic zinc modulated specific genes in the control group. Among these genes, Apid1 and Burs were positively modulated.

In the figure, ‘Apid1’ refers to the apidaecin1 gene, characterized by Casteel [33], which is related to a group of antibacterial peptides, and the main humoral components of hemolymph are induced in infections caused by bacteria. ‘Burs’ refers to the Bursicon gene, a neurohumoral agent responsible for cuticle pigmentation and wing expansion during insect metamorphosis [34]. Our transcriptome analysis indicated that zinc from inorganic sources positively modulated genes related to immunity and body development processes in *Apis mellifera* bees.

Among the downregulated genes previously studied, ATP5G2, Cpap3-d, Apd-1, Apd-2, Apd-3, TpnCIIb, and CPR27,thegenes of the Apid family were characterized by Kucharski [35], whose function is related to the production of cuticle proteins in *A. mellifera*. The Apd-1 gene can interfere with cuticular maturation in adults, Apd-2 is found in the internal cuticles (stomach and trachea), and Apd-3 is more widely expressed, which is related to cuticles and not pigmented.

ATP5G2 (NCBI) is related to the synthesis of ATP by mitochondria. TpnCIIb participates in the synthesis of troponin C, which is responsible for the muscle contraction mechanism [36] and CPR27 (NCBI) is a cuticular protein that is possibly related to the exoskeleton of these insects.

Concerning the referred biological processes, we observed that the inorganic zinc measurements mainly altered genes related to the arthropod body development process. As previously reported by Hiki [25], in crustaceans, zinc may have a direct relationship with the inhibition of the molting process. The process of energy synthesis (ATP) and muscle contraction mediated by troponin C also seems to be related, since at this stage the arthropod needs a large amount of energy to rupture the exoskeleton and undergoesits metamorphosis.

**Fig. 8:**
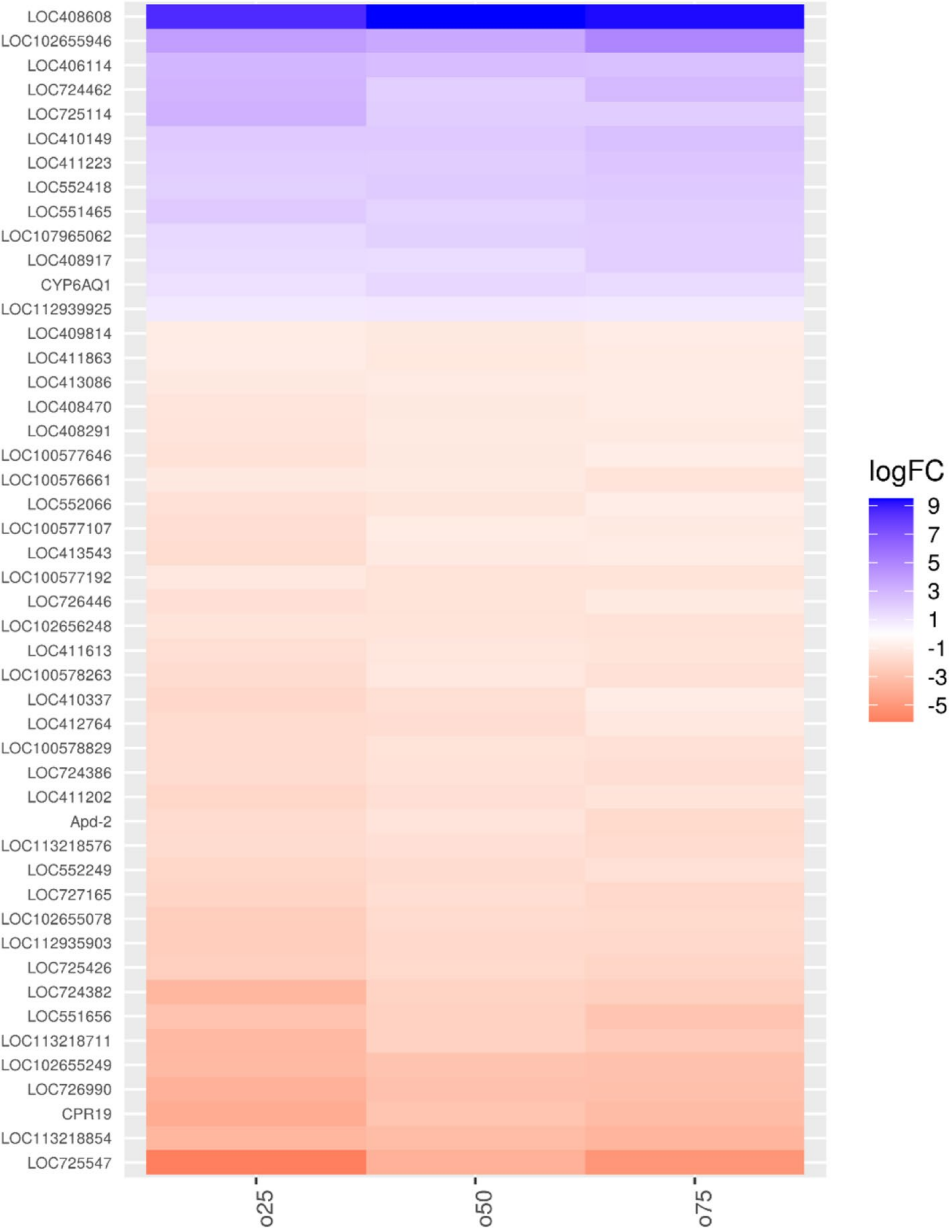
Clustering analysis of the differentially expressed genes between experimental groups supplemented with organic source of zinc and control groups

Regarding the groups supplemented with organic zinc (Fig. 8), we observed less enrichment of pathways when compared to treatments that received zinc from an inorganic source. However, some modulated genes that are common in the groups that received supplementation with an inorganic zinc source such as Apd-2 and CPR19, showed that the mineral zinc, regardless of the source, can affect metabolic pathways related to extremely important biological processes in *A. mellifera* honeybees.

## Conclusions

Our results indicate that zinc supplementation modulates the expression of many differentially expressed genes with an important role in the development of *Apis mellifera* bees, such as chitin binding and metabolic processes. All the information obtained from this study can contribute to future research in the field of bee nutrigenomics.

### Material and Methods

#### Local

The experiment was conducted at the apiary in the beekeeping area of the Lageado Experimental Farm, Faculty of Veterinary Medicine and Animal Science, UNESP, Botucatu, São Paulo, Brazil. It was conducted at the following geographic coordinates: 22° 49′ South and 48° 24′ West. The area is characterized by a humid subtropical climate with an average altitude of 623 m.

### Experimental Groups

Thirty-five beehives of Africanized *A. mellifera* were standardized for the number of breeding and feeding frames and distributed in four treatments (five hives per treatment):n0, control treatment without organic zinc supplementation; ZnO25, supplementation with 25 ppm organic zinc; ZnO50, supplementation with 50 ppm organic zinc; ZnO75, supplementation with 75 ppm organic zinc; ZnI25, supplementation with 25 ppm inorganic zinc; ZnI50, supplementation with 50 ppm inorganic zinc; ZnI75, supplementation with 75 ppm inorganic zinc.

All treatments were supplemented with sugar syrup. However, in the Zn0 treatment, there was no addition of organic zinc, while the other treatments received different concentrations of organic zinc, as described previously.

These values were based on the recommendation of Herbert and Shimanuki [18], who suggested the supplementation of 50 ppm of inorganic zinc.

The organic zinc source used was ferrous zinc methionine (16% zinc) and the inorganic source used was zinc sulfate, which was diluted in sugar syrup at a ratio of 1:1 (m/v) commercial crystal sugar and water, supplied using a Boardman feeder (500 mL per week) for one month. The levels provided were confirmed by atomic absorption spectroscopy (FAAS)for the organic and inorganic zinc levels. The following values were obtained: Zn0, 0.00 ppm; ZnO25, 23.16 ppm; ZnO50, 41.07 ppm; ZnO75, 67.44 ppm; ZnI25, 23.5 ppm; ZnI50, 49.4 ppm;ZnI75, 75.09 ppm.

### Bee Harvest

At the end of the experimental period, approximately 140 6-day-old bees from 35 hives were collected. For the collection, 12 frames with operculated brood areas were removed, one frame per hive, and then they were wrapped in tissue and placed in an incubator at 30 °C and humidity of 60% until the emergence of adults. The emerged workers were marked on the pronotum with a non-toxic pen, while they were attached to the frame. After being marked, the bees were reintroduced into their original hives, and smoke was used to disguise the colony recognition hydrocarbons that could hinder the acceptance of the returning bees. After six days, each bee was collected using an entomological tweezer and immediately stored in plastic pots in an ultra-freezer (− 80 °C) [37].

### Preparation and sequencing of the RNA-Seq library

Total RNA was extracted from the brain pools of *A. mellifera* nurse bees (20 brains per pool) using the TRIzol® reagent protocol according to the manufacturer's instructions (ThermoFisher Scientific, Waltham, USA) [38]. RNA concentrations were quantified in each sample using a QubitTM 2.0 fluorometer (ThermoFisher Scientific, Waltham, USA). The levels of RNA degradation were assessed using a 1% agarose gel. The cDNA libraries were constructed with 200 ng of total RNA using the SureSelect Strand Specific RNA Library Preparation Kit (Agilent Technologies, Santa Clara, USA), following the manufacturer's instructions. The products from the library were sequenced using an Illumina Nextseq platform (Illumina, San Diego, USA) in a single-bore run of 150 bp.

### RNA-Seq data processing and difference analysis of genetic expressions

The FASTQC program was used to check the adapter content and assess the quality of raw readings. Data alignment was performed with Burrows-Wheeler Aligner (BWA) v0.7.12, using Amel_HAv3.1 from NCBI (RefSeq assembly accession: GCF_003254395.2) as a reference. The feature count matrix was created using HTSeq v0.11.2 and the GTF annotation file from Amel_HAv3.1. Data analysis, visualization, and plotting were performed using RStudio for the R language, including the ggplot2 v3.3.2 package. Differential expression analysis was performed using the edgeR v3.30.3 package available at Bioconductor software project for the R language. Low expression genes were filtered by keeping genes that had the count per million greater than one in at least two of the samples. The counts were normalized using the TMM normalization. The negative binomial generalized log-linear model was used in the differential expression analysis, and the Benjamini & Hochberg procedure (FDR) was used for multiple testing corrections. Genes with an adjusted p-value < 0.05 were considered to be significantly regulated.

### Genetic ontology analysis

Gene ontology analysis was performed for the lists of genes identified with differential expression using the gprofiler2 package, an R interface to the g: Profiler tools (https://biit.cs.ut.ee/gprofiler/gost). Fisher's exact test p-values were adjusted using the Benjamini & Hochberg procedure (FDR) for multiple testing correction, and statistical significance was considered when the adjusted p-value was < 0.05. KEGG pathways database were obtained from KEGG (Kyoto Encyclopedia of Genes and Genomes, http://www.genome.jp/kegg/), and then the statistical enrichment of DEGs in KEGG pathways was determined using gprofiler2 package for R [39].

## Supplementary Information

**Additional file 1: Supplementary Material. Table S1.** Statistics of differential gene expression sequencing. **Figure S1.** Pairwise Pearson correlation coefficients (Rho) of global expression values. **Figure S2.** Gene ontology analysis of both experimental group’s intersection. Red color indicates biological processes and blue color indicates KEGG [40–42].

## Data Availability

The datasets generated and/or analysed during the current study are available in the NCBI Sequence Read Archive (SRA) repository, https://dataview.ncbi.nlm.nih.gov/object/PRJNA730561, BioProjectID PRJNA730561.
