# Supplementary material for: Zinc supplementation modifies brain tissue transcriptome of Apis mellifera honeybees
Source: BMC Genomics. 2022 Apr 8;23:282. doi: 10.1186/s12864-022-08464-1 (PMC8994358; doi:10.1186/s12864-022-08464-1)
Supplement: Supplementary file 1 — Additional file 1: Supplementary Material. Table S1. Statistics of differential gene expression sequencing. Figure S1. Pairwise Pearson correlation coefficients (Rho) of global expression values. Figure S2. Gene ontology analysis of both experimental group’s intersection. Red color indicates biological processes and blue color indicates KEGG [40–42]. [file 12864_2022_8464_MOESM1_ESM.docx]

**Supplementary Material**

**Table S1.** Statistics of differential gene expression sequencing.

| Sample | Alignment_not_unique | Ambiguous | Feature | No_feature | Not_aligned | Too_low_aQual |
| --- | --- | --- | --- | --- | --- | --- |
| Znctl-1 | 0% (0) | 0,8% | 60.9% | 7.9% | 1% | 29.4% |
| Znctl-2 | 0% (0) | 0.3% | 24% | 2.5% | 43.3% | 29.9% |
| Znctl-3 | 0% (0) | 0.8% | 69.9% | 4.5% | 1.1% | 23.7% |
| Znclt-4 | 0% (0) | 1.1% | 86.9% | 7.9% | 1% | 3.1% |
| ZnI25-1 | 0% (0) | 0.8% | 67% | 6.2% | 1% | 25% |
| ZnI25-2 | 0% (0) | 1.1% | 80.3% | 11.1% | 1.4% | 6.1% |
| ZnI25-3 | 0% (0) | 1% | 82.2% | 8.6% | 3.2% | 5% |
| ZnI25-4 | 0% (0) | 1% | 79.8% | 11.9% | 2.1% | 5.2% |
| ZnI50-1 | 0% (0) | 0.9% | 79.5% | 4.6% | 1% | 14% |
| ZnI50-2 | 0% (0) | 1% | 88.5% | 4.6% | 0.6% | 5.3% |
| ZnI50-3 | 0% (0) | 0.9% | 85.6% | 6.4% | 1.5% | 5.5% |
| ZnI50-4 | 0% (0) | 0.9% | 83.1% | 7.6% | 0.8% | 7.5% |
| ZnI75-1 | 0% (0) | 1% | 85.1% | 5.4% | 0.8% | 7.8% |
| ZnI75-2 | 0% (0) | 0.9% | 77.9% | 3.6% | 11.4% | 6% |
| ZnI75-4 | 0% (0) | 0.8% | 78.8% | 5.9% | 8% | 6.4% |
| ZnO25-1 | 0% (0) | 0.8% | 75.5% | 4.4% | 0.7% | 18.6% |
| ZnO25-2 | 0% (0) | 0.8% | 77.6% | 5% | 2.8% | 14.7% |
| ZnO25-3 | 0% (0) | 0.7% | 82.6% | 4.2% | 1.2% | 11.6% |
| ZnO25-4 | 0% (0) | 1% | 75.3% | 3.9% | 0.9% | 17.3% |
| ZnO50-1 | 0% (0) | 1% | 75.9% | 4.1% | 0,9% | 17.8% |
| ZnO50-2 | 0% (0) | 1.2% | 85.1% | 7% | 9.4% | 6.1% |
| ZnO50-3 | 0% (0) | 0.7% | 86.5% | 4.7% | 1.8% | 6.6% |
| ZnO50-4 | 0% (0) | 1.1% | 68.7% | 10.6% | 2.3% | 10.5% |
| ZnO75-1 | 0% (0) | 1% | 85.2% | 6% | 1.4% | 5.8% |
| ZnO75-2 | 0% (0) | 0.9% | 83% | 7.7% | 1.6% | 6% |
| ZnO75-3 | 0% (0) | 0.9% | 80.8% | 9.1% | 2.3% | 7.8% |
| ZnO75-4 | 0% (0) | 0.9% | 80.9% | 4.1% | 1.6% | 12.6% |


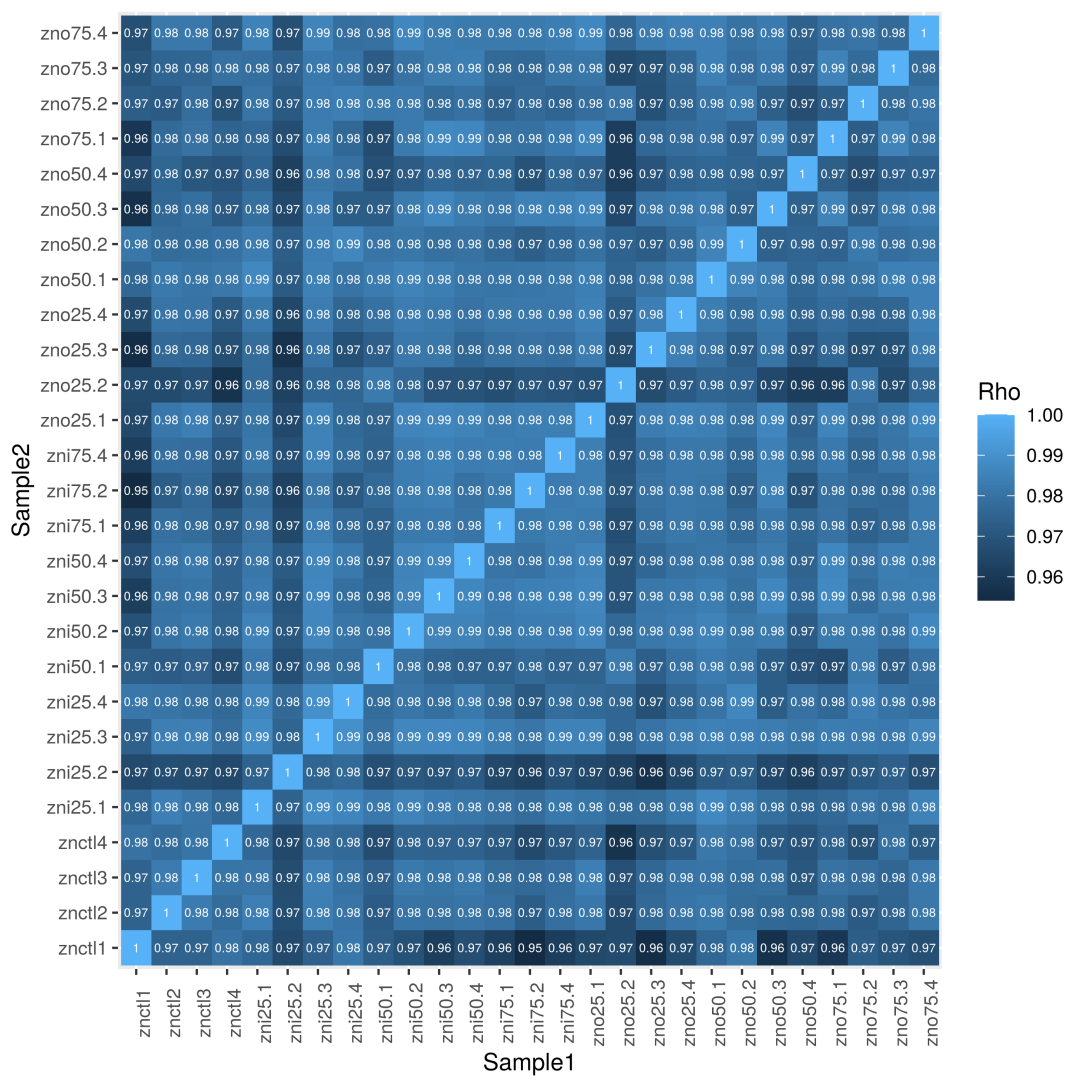


**Figure S1.** Pairwise Pearson correlation coefficients (Rho) of global expression values.


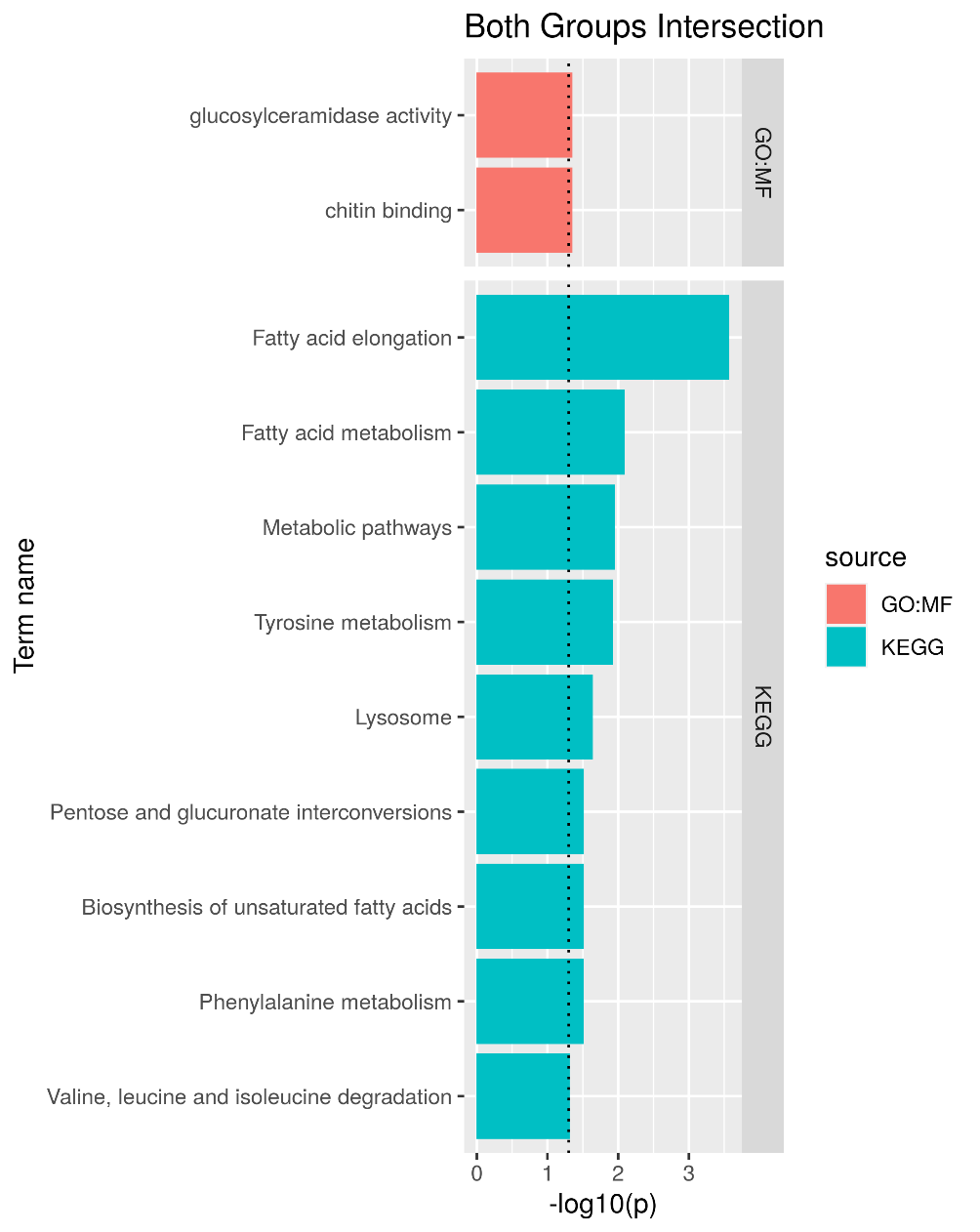


**Figure S2.** Gene ontology analysis of both experimental group’s intersection. Red color indicates biological processes and blue color indicates KEGG [40, 41, 42].
